# The Complexity of Burnout Experiences among Care Aides: A Person-Oriented Approach to Burnout Patterns

**DOI:** 10.3390/healthcare11081145

**Published:** 2023-04-17

**Authors:** Yinfei Duan, Yuting Song, Trina E. Thorne, Alba Iaconi, Peter G. Norton, Carole A. Estabrooks

**Affiliations:** 1 Faculty of Nursing, University of Alberta, Edmonton, AB T6G 1C9, Canada; yuting.song@ualberta.ca (Y.S.); tethorne@ualberta.ca (T.E.T.); alba.iaconi@ualberta.ca (A.I.); cestabro@ualberta.ca (C.A.E.); 2 School of Nursing, Qingdao University, Qingdao 266071, China; 3 Department of Family Medicine, University of Calgary, Calgary, AB T2N 4N1, Canada; norton@ucalgary.ca

**Keywords:** burnout, care aides, nursing homes, long-term care, quality of work-life, person-oriented approach, latent profile analysis

## Abstract

Care aides working in nursing homes experience burnout attributed to various workplace stressors. Burnout dimensions (exhaustion, cynicism, and reduced professional efficacy) interact to form distinct burnout patterns. Using a person-oriented approach, we aimed to identify burnout patterns among care aides and to examine their association with individual and job-related factors. This was a cross-sectional, secondary analysis of the Translating Research in Elder Care 2019–2020 survey data collected from 3765 care aides working in Canadian nursing homes. We used Maslach Burnout Inventory to assess burnout and performed latent profile analysis to identify burnout patterns, then examined their associations with other factors. We identified an *engaged* pattern (43.2% of the care aide sample) with low exhaustion and cynicism and high professional efficacy; an *overwhelmed but accomplished* pattern (38.5%) with high levels of the three dimensions; two intermediate patterns—a *tired and ineffective* pattern (2.4%) and a *tired but effective* pattern (15.8%). The *engaged* group reported the most favorable scores on work environment, work-life experiences, and health, whereas the *tired and ineffective* group reported the least favorable scores. The findings suggest complex experiences of burnout among care aides and call for tailored interventions to distinct burnout patterns.

## 1. Introduction

Unregulated workers (health care aides, personal support workers, certified nurse assistants) constitute the largest workforce in nursing homes [1,2,3]. These workers experience a high risk of burnout, which threatens the quality of their work-life and which can subsequently affect the quality of care [1,2,3,4]. Burnout is defined as a prolonged response to chronic emotional and interpersonal stressors on the job and can be characterized by three dimensions: exhaustion (feelings of emotional and physical depletion), cynicism (a negative detached response to the job), and reduced professional efficacy (feelings of incompetence and a lack of achievement at work) [5].

Burnout among care aides has not been studied as widely as burnout among other health care worker groups in nursing homes [4]. The few studies that do exist have taken a variable-oriented approach [6] and assessed each dimension as a discrete variable [7,8,9,10]. However, this approach overlooks the dynamics among burnout dimensions and their configurations within individuals [11]. Therefore, a person-oriented approach (or person-centered approach) can address the interactions among the burnout dimensions and allow for the assessment of an individual’s burnout experience as a whole [6,12,13].

The potential of having variations of the three burnout dimensions that combine in ways to form burnout patterns has been suggested in several theoretical models of burnout [14,15,16,17]. Leiter and Maslach (1988) proposed that exhaustion occurred first as a response to work-related stressors; high exhaustion would lead individuals to withdraw themselves psychologically from their work (cynicism), which would eventually lead to diminished professional efficacy [15]. Lee and Ashforth (1993) argued that elevated exhaustion evoked a direct decrease in professional efficacy rather than indirectly through cynicism [16]. Alternatively, Golembiewski and others (1986), in their phase model, hypothesized that professional detachment (cynicism) developed first, followed by reduced perception of accomplishment (professional efficacy) and increased exhaustion [14]. Despite inconsistent views on the causal ordering of burnout dimensions, these models agree that the three burnout dimensions are not always concurrent, nor do they constitute a single, one-dimensional phenomenon. Accordingly, we hypothesized that a cross-sectional examination of three burnout dimensions with a person-oriented approach might reveal various burnout patterns among individuals.

The person- oriented approach has not been applied to care aides, despite an increase in its use in other populations. This hinders a deeper understanding of how burnout develops among this particular workforce. Limited knowledge of distinct burnout patterns will impede the development of tailored interventions to reduce burnout among care aides. In this study, we sought to explore the dynamics among burnout dimensions and how they collectively shape experiences of burnout within care aides. We aimed to (1) identify burnout patterns among care aides in nursing homes and (2) examine the associations of various individual and job-related factors with these burnout patterns. The findings of this study are hoped to offer insights into the complexity of burnout experiences in care aides and to provide practical implications for the development of burnout reduction interventions that best benefit this population.

## 2. Materials and Methods

### 2.1. Data Source and Sample

This study is associated with the Translating Research in Elder Care (TREC) program. TREC is a long-term, multi-project research program to investigate factors influencing the quality of care for older adults in nursing homes and the quality of work-life for staff [18]. We used care aide survey data collected between September 2019 and February 2020 from 91 nursing homes in urban areas within the Canadian provinces of Alberta, British Columbia, and Manitoba. Nursing homes were selected using random, stratified, proportional sampling [18]. The strata comprised region, facility size, and facility ownership model [18]. The sampled nursing homes are a representation of nursing homes across the three participating provinces, with varying sizes (large with >120 beds, medium with 80–120 beds, and small with 35–79 beds), owner-operator model (public not-for-profit, voluntary not-for-profit, and private for profit).

Care aides were invited to complete the survey if they had worked in the same nursing home unit for 3 or more months, with a minimum of 6 shifts per month [18]. The care aide survey data were collected using a computer-assisted personal interview (CAPI) format. Trained interviewers went to participating nursing homes to administer structured CAPI survey interviews to individual care aides in a private and quiet space. During the interview, the interviewer read survey questions to the respondent using standardized language and instructed the respondents to refer to color coded cards outlining response options [18]. A total of 3765 care aides (response rate 70%) were included in the analysis. 

### 2.2. Variables and Measures

We used the short form of the Maslach Burnout Inventory-General Survey (MBI-GS), which has 3 scales measuring exhaustion, cynicism, and professional efficacy [19]. Each scale of the short form of the MBI-GS has 3 items measured on a 7-point Likert frequency-based scale (0 = never; 1 = a few times a year or less; 2 = once a month or less; 3 = a few times a month; 4 = once a week; 5 = a few times a week; 6 = daily). A composite score for each scale was derived by taking the mean of the scale items. A high risk of burnout was reflected in high scores on exhaustion and cynicism and low scores on professional efficacy. A 3-factor structure for the original MBI-GS (factorial validity) was examined with various occupational groups [19,20,21]. The internal consistency of each of these scales was satisfactory, with Cronbach’s alpha being 0.84–0.90 for exhaustion, 0.74–0.84 for cynicism, and 0.70–0.78 for professional efficacy [22]. Cronbach’s alpha, in the current sample, was 0.74 for exhaustion, 0.64 for cynicism, and 0.51 for professional efficacy.

We examined a range of individual and job-related factors, suggested by previous research, that may be associated with burnout patterns [7]. These included care aide demographics, perceptions of work environment (measured with the Alberta Context Tool [23]), work-life experiences (adequate job orientation, experiencing residents’ responsive behaviors, rushed or missed care [24], job satisfaction (measured with an adapted version of the Michigan Organizational Assessment Questionnaire Job Satisfaction Subscale) [25], psychological empowerment (measured with an adapted version of Spreitzer’s Psychological Empowerment Measure) [26,27], work engagement (measured with an adapted version of the Utrecht Work Engagement Scale—9 items) [27,28], change-oriented organizational citizenship behaviors (measured with Choi’s 4-item Change-Oriented Organizational Citizenship Behaviors) [27,29], and physical and mental health (measured with the Short Form 8 Health Survey (SF-8) [30]. A detailed description of measures for each variable is presented in Table S1 in the Supplementary Materials.

### 2.3. Analysis

We used latent profile analysis (LPA) to identify burnout patterns. LPA identifies a latent categorical variable that divides a sample into mutually exclusive and exhaustive sub-groups, with each representing a unique latent profile [31]. The composite scores of the 3 burnout scales were used as observed indicators to fit a set of LPA models with different numbers of profiles using Mplus 7.0 [32]. We used various model fit indices to determine the ideal number of profiles that demonstrated the best fit to the data, including the Akaike information criterion; the Bayesian information criterion (BIC); sample-size adjusted BIC; the Lo, Mendell, and Rubin test; and entropy (measuring classification uncertainty) [31]. We also considered conceptual meaning, interpretability of profiles, and model parsimony in the model selection process.

We conducted a series of post hoc analyses to examine the association of profile membership (the care aide’s most likely profile based on the posterior probabilities) with individual and job-related factors. The post hoc approach is justified when the entropy value is 0.80 or above [33]. We used analysis of variance (ANOVA) and chi-square tests to compare the demographics of care aides by profile membership. We used 3-level random intercept regression to compare job-related variables (perceptions of work environment, work-life experiences, and health status) across profile membership, controlling for the clusters of care aides nested in the same unit and facility. Stata 16 was used for post hoc analyses [34].

## 3. Results

### 3.1. Four Burnout Patterns

**Figure 1:**
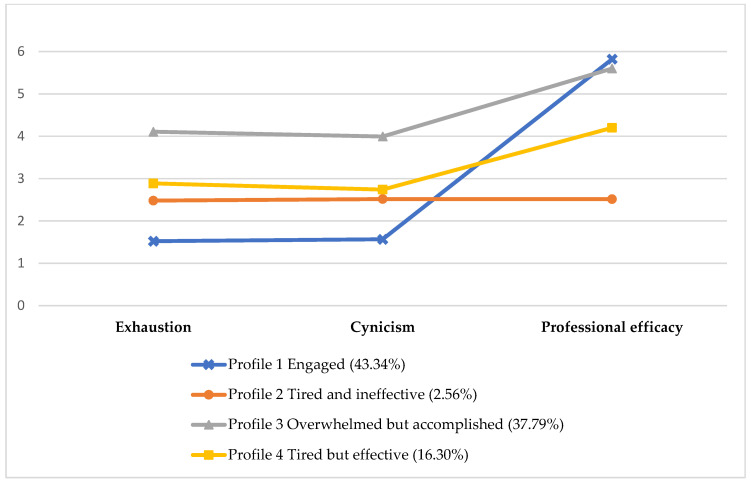
Estimated means of burnout scores by latent profile based on the 4-profile model.

Care aides in our sample scored on average 2.75, 2.70, and 5.39 on exhaustion, cynicism, and professional efficacy, respectively (range 0–6). Table 1 compares model fit indices of the LPA models with different numbers of profiles. The four-profile model was selected based on the predefined model selection criteria. A detailed description of the model building and selection processes is presented in Table S2 in the Supplementary Materials, while burnout scores by profile are presented in Figure 1 and Table 2.

The most frequent profile (43.2% of the sample) was characterized by low levels of exhaustion (1.47) and cynicism (1.50), and a high level of professional efficacy (5.82). We labeled this profile *engaged*. By comparison, the *overwhelmed but accomplished* profile (38.5%) comprised high levels of exhaustion (4.15), cynicism (4.50), and professional efficacy (5.60).

The remaining two profiles reported comparable levels of exhaustion (2.53–2.85) and cynicism (2.55–2.71), but differed in professional efficacy. The profile reporting a medium level of professional efficacy (2.45) was labeled *tired and ineffective* (less than 2.4% of the sample), and the profile reporting moderately high efficacy (4.15) was labeled *tired but effective* (15.8%).

### 3.2. Differences in Demographics and Job-Related Characteristics by Burnout Pattern

Table 3 shows the demographics of care aides by burnout pattern. The *engaged* (73%) and *overwhelmed but accomplished* (70%) groups each had a higher proportion of care aides aged 40 years or older compared with the *tired but effective* (62%) group. The *engaged* (77%) and *overwhelmed but accomplished* (80%) groups each had a higher proportion of care aides born outside of Canada than the *tired but effective* (70%) group. The *overwhelmed but accomplished* (72%) group had a higher proportion of care aides who spoke English as an additional language than the *engaged* (67%) and *tired but effective* (63%) groups. The *engaged* (12.3 ± 9.4) and *tired and ineffective* (14.12 ± 10.5) groups reported more years worked as a care aide than the *tired but effective* (11.06 ± 7.8) group.

Table 4 presents cross-profile comparisons in a range of job-related variables including care aides’ perceptions of work environment, work-life experiences, and health status. The *engaged* group consistently reported the most positive scores on these variables, whereas the *tired but effective* and/or *tired and ineffective* groups reported the most negative scores. The *overwhelmed but accomplished* group was midway between for certain job-related variables, including psychological empowerment, work engagement, and adequate job orientation. However, the *overwhelmed but accomplished* and the *engaged* groups were similar with regard to positive perceptions of communication (i.e., the Alberta Context Tool scales of formal and informal interactions) and organizational citizenship behaviors, while the *overwhelmed but accomplished* reported negative results comparable to the *tired but effective* and *tired and ineffective* groups on other variables such as perceptions of organizational slack in staffing, job satisfaction, self-reported health, experiences of responsive behaviors from residents, and rushing/missing care.

## 4. Discussion

### 4.1. Complex Relationships among Burnout Dimensions

We identified four burnout patterns that represent distinct configurations of burnout dimensions among care aides. While previous variable-oriented studies indicated that, on average, the three burnout dimensions rise and fall together [4,35], our findings revealed more complex relationships among the dimensions. Overall, we observed that exhaustion and cynicism jointly formed three patterns representing three general levels of burnout (high, medium, and low), while the inclusion of reduced professional efficacy resulted in four more nuanced patterns. The burnout patterns may reflect complex developmental sequence and causal links among the three dimensions, as hypothesized in the theoretical models of burnout (Leiter and Maslach (1988) [15]; Golembiewski et al. (1986) [14]; and Lee and Ashforth (1993) [16]). However, none of these models fully explained interactions among the three burnout dimensions underlying the burnout patterns in this care aide sample.

Consistent with other empirical studies that apply person-oriented methods with other groups of heath care workers [36,37,38,39], we identified the *engaged* pattern as the most prevalent pattern (accounting for 43% of the care aide sample). Similar to the traditional engagement–burnout continuum defined by Leiter and Maslach (1998) [40], the *engaged* pattern represents one end point of the continuum, characterizing individuals with relatively positive work experiences. People exhibiting an *engaged* pattern have not developed burnout symptoms or are at an early stage of burnout.

The Leiter and Maslach (1998) model [40] proposed an opposite pattern that represents the other end point of the engagement–burnout continuum, where individuals demonstrate extremely high levels of exhaustion and cynicism and an extremely low level of professional efficacy [14,15,16]. Other burnout models, including the phase model by Golembiewski et al. (1986) [14] and that of Lee and Ashforth (1993) [16], also predict similar patterns that represent two extremes. However, although we identified a positive *engaged* pattern, an opposing pattern was absent in our sample. This is inconsistent with studies in other health care occupational groups such as physicians and nurses, in which a negative pattern was identified along with a positive one [6,36,37]. Our findings indicate that the negative end point or the late stage of burnout experience for care aides may not produce all three burnout dimensions.

The other three patterns (*overwhelmed but accomplished*, *tired but effective*, and *tired and ineffective* which accounted for 38%, 16%, and 2% of the care aide sample, respectively) further suggest complex relationships among the three burnout dimensions, especially the relationship of reduced professional efficacy with exhaustion and cynicism. We observed that exhaustion and cynicism covaried in a somewhat linear manner, while reduced professional efficacy demonstrated a non-linear relationship with them. Similar findings were reported in previous studies [36,37,38,39]. The covariation of exhaustion and cynicism may result from a causal relationship. According to the models of Leiter and Maslach (1988) [15] and Lee and Ashforth (1993) [16], alongside other empirical studies [17], cynicism is the immediate consequence of exhaustion in that psychologically detaching oneself from work is often used as a (dysfunctional) coping strategy when experiencing exhaustion. This is especially true for occupations with high rates of human interaction, such as health care workers. Whether this causal assumption applies to care aides will need further investigation. 

The distinct role of professional efficacy in forming burnout patterns also deserves further research. On the one hand, the *overwhelmed but accomplished* group maintained a high level of professional efficacy, although they had experienced high levels of exhaustion and cynicism. On the other hand, the *tired and ineffective* group experienced the lowest level of professional efficacy compared with the other three groups, although they reported intermediate levels of exhaustion and cynicism. Leiter and Maslach’s model (1988) [15] proposed that exhaustion occurs first, leads to cynicism, and finally reduces professional efficacy. In this regard, an *overwhelmed but accomplished* pattern might represent individuals who had experienced exhaustion and cynicism but had yet to develop reduced professional efficacy. However, our findings suggest that the sequential occurrence of the three burnout dimensions, as suggested by Leiter and Maslach (1988) [15], did not apply to all the patterns (particularly *tired and ineffective*). More empirical research is encouraged to theorize the relationship of reduced professional efficacy with exhaustion and cynicism.

Measurement issues may be another explanation for the linear relationship between exhaustion and cynicism and their non-linear relationship with professional efficacy. Schaufeli and Salanova (2007) [41] suggested that a stronger association between scores of exhaustion and cynicism may be a result of measurements using items framed negatively, whereas items of professional efficacy are framed positively [42,43]. 

#### Theoretical Implications

Although exploratory, our findings point to several directions for future research to advance a more complete and nuanced understanding of burnout in care aides. Specifically, we suggest further investigations around conceptual distinctiveness, developmental sequence, and causal connectedness among the three burnout dimensions. Qualitative research or longitudinal quantitative methods will be useful in this regard. In particular, longitudinal transition analysis, a person-oriented approach to identify within-individual transitions between burnout patterns over time, will provide theoretical implications to consider whether the burnout patterns identified through a cross-sectional analysis fall under one developmental model, or if each pattern is explained by a unique developmental or causal model [44]. Future research may produce new theoretical models of burnout and measurement tools that are specific to the care aide population, which is necessary if care aides exhibit unique burnout experiences.

### 4.2. Associations of Burnout Patterns with Work Environment and Quality of Work-Life

We observed that care aides’ perceptions of work environment and work-life experiences differed significantly across burnout patterns, suggesting that the identified burnout patterns are meaningful with respect to their capacity to reflect or predict other theoretical constructs such as work environment and work experiences. The *engaged* group, unsurprisingly, reported the most positive results on all job-related variables, whereas the *tired but effective* and, most notably, the *tired and ineffective* groups demonstrated the most negative results. 

The *overwhelmed but accomplished* and *engaged* groups, both of which reported a high level of professional efficacy, shared similar positive perceptions of communication and exhibition of organizational citizenship behaviors. This finding indicates that professional efficacy might be uniquely associated with these areas of work-life. Likewise, since the *overwhelmed but accomplished* group (despite high professional efficacy) reported similar results to the *tired but effective* and *tired and ineffective* groups on particular variables (including low job satisfaction, poor self-reported health, high frequencies of experiencing responsive behaviors from residents, and rushing/missing care), it is reasonable to speculate that exhaustion and cynicism are jointly associated with these areas of work-life. Our findings lend some support to previous literature, which suggested each burnout dimension could be closely associated with certain areas of work-life [4,7,35,36,45,46,47,48]. Nevertheless, we call for more research with a focus on examining the causal relationships of the burnout patterns to work environment elements and work-life experiences.

#### Practical Implications

Although early and exploratory, our research suggests that it may be more appropriate and effective to have burnout-reduction efforts tailored to distinct burnout patterns. For example, preventive interventions would benefit the *engaged* group, while more treatment-oriented interventions may be needed for the other three groups; reducing exhaustion and cynicism could be the focus of intervention for the *overwhelmed but accomplished* group, while improving professional efficacy could be prioritized for the *tired and ineffective* group. Our findings provide clues regarding possible intervention targets. For example, intervention for the *overwhelmed but accomplished* group may need to target work environment elements related to workload, resources, and training on dementia care. Extra efforts to modify other aspects of the work environment, such as teamwork, communication, and adequate job orientation, may be beneficial for the *tired but effective* and *tired and ineffective* groups.

### 4.3. Limitations

Some important limitations must be noted. First, LPA is largely exploratory and descriptive. Although we applied various model fit criteria in model selection, the validity of the burnout patterns could not be completely established. For example, we could not conclusively determine if the *tired and ineffective* group was a spurious profile due to response bias; this group tended to endorse middle options on the burnout scales. Despite this, we treated it as a meaningful pattern given its distinguishing features on other individual and job-related factors. Challenges to the validity of burnout patterns may also come from the low to moderate internal consistency of the scales of cynicism and professional efficacy, or potential issues encountered in survey research such as social desirability bias and recall bias [49]. The cross-sectional design precludes interpreting the results as developmental patterns of burnout experiences, or asserting causal relationships between burnout patterns and job-related factors. Our findings may only be generalized to care aides working in urban nursing homes in western Canada prior to the COVID-19 pandemic. The pandemic may have deteriorated and complicated burnout experiences of care aides, possibly contributing to more complex burnout patterns [50]. Future research can expand to examine burnout patterns among care aides during or post-pandemic and compare if burnout patterns shift in a significant manner.

## 5. Conclusions

Using LPA in this exploratory study, we identified four burnout patterns among nursing home care aides, providing an improved understanding of the range of work experiences they face. The burnout patterns are significantly associated with various individual and job-related factors, findings that may assist the development of burnout reduction interventions tailored to distinct burnout patterns. Future research on the developmental sequence and causality among the three burnout dimensions is needed to fully interpret the burnout patterns.

## Figures and Tables

**Table 1:** Model fit for latent profile analysis models with different numbers of latent profiles (*n* = 3765).

Model	Number of Free Parameters	Log Likelihood	AIC	BIC	Sample-Size Adjusted BIC	LMR Test *p*-Value ^a^	Entropy	Latent Profile Probabilities (Range)	Smallest Class %
3 profiles	14	−17,738.65	35,505.31	35,592.58	35,548.09	<0.001	0.794	0.90–0.91	13.69
4 profiles	18	−17,445.20	34,926.39	35,038.60	34,981.40	<0.002	0.829	0.89–0.91	2.56
5 profiles	22	−17,099.82	34,243.63	34,380.77	34,310.87	0.056	0.875	0.84–0.99	2.43
6 profiles	26	−16,782.44	33,616.87	33,778.94	33,696.33	0.002	0.908	0.86–1.00	1.06
7 profiles	30	−16,762.78	33,585.55	33,772.56	33,677.23	0.012	0.877	0.87–0.99	2.41

Notes: In fitting each latent profile analysis model, we allowed free estimation of profile-specific indicator means with error variances of indicators constrained to be equal across profiles. Within-profile residual covariances were constrained to be zero, thereby assuming three burnout dimension indicators are independent within-profile. The clustering effect at the facility level was controlled for in computing standard errors and chi-square tests of model fit. For missing data, Mplus uses full-information maximum likelihood to estimate cases with missing values on some (not all) variables and removes cases with missing values on all variables. In our study, zero cases had missing values on all three burnout scores and the number of missing cases was 9, 19, and 4 for the scale of exhaustion, cynicism, and professional efficacy, respectively. ^a^: LMR likelihood ratio test compares the current model (k profiles) to a model with k-1 profiles. Statistical significance indicates that the current model (k profiles) is a better fit than the model with k-1 profiles. AIC = Akaike information criterion; BIC = Bayesian information criterion; LMR test = Lo–Mendell–Rubin test.

**Table 2:** Burnout scores and individual items by profile membership ^a^ (*n* = 3765).

	Profile 1 Engaged (43.24%)	Profile 2 Tired and Ineffective (2.42%)	Profile 3 Overwhelmed but Accomplished (38.54%)	Profile 4 Tired but Effective (15.80%)	F (ANOVA)	*p*
	Mean (SD)	Mean (SD)	Mean (SD)	Mean (SD)		
**Exhaustion composite score ^b^**	1.47 (1.16)	2.53 (1.79)	4.15 (1.07)	2.85 (1.53)	1253.31	<0.001
I feel tired when I get up in the morning ^c^	1.77 (1.82)	2.68 (1.97)	4.17 (1.60)	3.26 (1.88)	488.76	<0.001
Working all day is really a strain ^b^	1.36 (1.80)	2.60 (2.20)	4.43 (1.58)	2.67 (2.00)	773.74	<0.001
I feel burned out from my work ^b^	1.26 (1.49)	2.30 (2.16)	3.85 (1.70)	2.60 (1.88)	626.55	<0.001
**Cynicism composite score ^b^**	1.50 (1.09)	2.55 (1.66)	4.05 (1.15)	2.71 (1.40)	1185.75	<0.001
I just want to do my job and not be bothered ^b^	3.20 (2.56)	3.71 (2.34)	5.15 (1.39)	3.85 (2.07)	224.54	<0.001
I have become more cynical about whether my work contributes anything ^b^	0.66 (1.33)	2.07 (2.14)	3.51 (1.96)	2.06 (1.90)	706.27	<0.001
I have become less enthusiastic ^b^	0.64 (1.20)	1.87 (1.93)	3.49 (1.85)	2.23 (1.84)	810.01	<0.001
**Professional efficacy composite score ^c^**	5.82 (0.31)	2.45 (0.61)	5.60 (0.42)	4.15 (0.41)	4775.3	<0.001
In my opinion, I am good at my job ^c^	5.95 (0.24)	4.59 (1.85)	5.84 (0.41)	5.45 (0.96)	256.97	<0.001
I feel exhilarated when I accomplish something at work ^c^	5.84 (0.46)	1.41 (1.48)	5.54 (0.74)	3.53 (1.77)	1487.55	<0.001
I have accomplished many worthwhile things in this job ^c^	5.67 (0.69)	1.35 (1.24)	5.41 (0.78)	3.46 (1.66)	1296.43	<0.001

Notes: We used listwise deletion to handle missingness. Missing cases range from 1 to 19 cases for the variables examined. ^a^: The profile membership was the care aide’s most likely profile based on the posterior probabilities. ^b^: Bonferroni multiple-comparison tests indicated significant differences in all comparisons except for the comparison between Profile 2 and 4. ^c^: Bonferroni multiple-comparison tests indicated significant differences in all comparisons.

**Table 3:** Demographic characteristics by profile membership ^a^ (*n* = 3765).

	Profile 1 Engaged (43.24%)	Profile 2 Tired and Ineffective (2.42%)	Profile 3 Overwhelmed but Accomplished (38.54%)	Profile 4 Tired but Effective (15.80%)	Chi^2^	Multiple Comparison with *p* < 0.008 ^b^
	Freq (%)	Freq (%)	Freq (%)	Freq (%)		
Age					47.03 ***	PF 4 vs. PF 1, PF 3
<30 years	111 (6.82)	12 (13.19)	119 (8.20)	76 (12.77)		
30–39 years	326 (20.02)	17 (18.68)	320 (22.05)	152 (25.55)		
40–49 years	518 (31.82)	25 (27.47)	455 (31.36)	188 (31.60)		
50–59 years	470 (28.87)	22 (24.18)	406 (27.98)	121 (20.34)		
> = 60 years	203 (12.47)	15 (16.48)	151 (10.41)	58 (9.75)		
Female	1452 (89.24)	83 (91.21)	1285 (88.62)	539 (90.74)	2.34	none
Born outside of Canada	1247 (76.60)	66 (72.53)	1160 (79.94)	417 (70.08)	24.05 ***	PF 4 vs. PF 1, PF 3
English as a second language	1092 (67.08)	56 (61.54)	1046 (72.09)	377 (63.36)	19.36 ***	PF 3 vs. PF 1, PF 4
Working in 2 or more NHs	389 (23.89)	29 (32.22)	355 (24.48)	142 (23.87)	3.30	none
Shift worked most					8.69	none
Day shift	803 (49.32)	40 (43.96)	769 (53.00)	297 (49.92)		
Evening shift	607 (37.29)	40 (43.96)	519 (35.77)	215 (36.13)		
Night shift	218 (13.39)	11 (12.09)	163 (11.23)	83 (13.95)		
Facility Size					6.39	none
Small (<80 beds)	193 (11.86)	5 (5.49)	195 (13.44)	77 (12.94)		
Med (80–120 beds)	507 (31.14)	28 (30.77)	448 (30.88)	181 (30.42)		
Large (>120 beds)	928 (57)	58 (63.74)	808 (55.69)	337 (56.64)		
Ownership					4.52	none
Public not for profit	329 (20.21)	16 (17.58)	288 (19.85)	115 (19.33)		
Voluntary not for profit	634 (38.94)	36 (39.56)	531 (36.6)	242 (40.67)		
Private for profit	665 (40.85)	39 (42.86)	632 (43.56)	238 (40.00)		
Health Region					62.51 ***	PF 3 vs. PF 1, PF 4
Alberta Health Edmonton Zone	402 (24.69)	28 (30.77)	299 (20.61)	165 (27.73)		
Alberta Health Calgary Zone	297 (18.24)	16 (17.58)	266 (18.33)	117 (19.66)		
British Columbia Interior Health Authority	194 (11.92)	9 (9.89)	110 (7.58)	67 (11.26)		
British Columbia Fraser Health Authority	453 (27.83)	13 (14.29)	446 (30.74)	125 (21.01)		
Winnipeg Regional Health Authority	282 (17.32)	25 (27.47)	330 (22.74)	121 (20.34)		
	**Mean (SD)**	**Mean (SD)**	**Mean (SD)**	**Mean (SD)**	**F**	**Multiple Comparison with *p* < 0.008 ^b^**
Years worked as a care aide	12.27 (9.40)	14.12 (10.53)	11.85 (8.79)	11.06 (7.84)	4.50 **	PF 4 vs. PF 1, PF 2
Years worked on the current unit	6.09 (6.34)	7.81 (8.62)	6.16 (6.11)	5.96 (6.01)	2.36	none

Notes: We used listwise deletion to handle missingness. Missing cases range from 0 to 3 for the variables examined. PF = profile. ^a^: The profile membership was the care aide’s most likely profile based on the posterior probabilities. ^b^: Bonferroni corrections were used in the multiple comparisons and adjusted *p* < 0.008 were used to indicate statistical significance. *** *p* < 0.001, ** *p* < 0.01.

**Table 4:** Perceptions of work environment, experiences of work-life, and health status by profile membership (*n* = 3765) ^a^.

	Profile 1 Engaged (43.24%)	Profile 2 Tired and Ineffective (2.42%)	Profile 3 Overwhelmed but Accomplished (38.54%)	Profile 4 Tired but Effective (15.80%)	Multiple Comparison with *p* < 0.008 ^b^
	Mean (SD)	Mean (SD)	Mean (SD)	Mean (SD)	
**Work environment**					
Leadership (1–5)	4.08 (0.55)	3.70 (0.71)	3.92 (0.55)	3.86 (0.56)	PF 1 > PFs 3, 4 > PF 2
Culture (1–5)	4.19 (0.47)	3.83 (0.60)	4.00 (0.52)	3.92 (0.52)	PF 1 > PF 3 > PFs 2, 4
Evaluation (1–5)	3.87 (0.59)	3.58 (0.74)	3.76 (0.60)	3.63 (0.62)	PF 1 > PF 3 > PFs 2, 4
Formal interactions (0–4)	1.50 (0.78)	1.46 (1.01)	1.50 (0.83)	1.40 (0.82)	PFs 1, 3 > PF 4
Informal interactions (0–9)	4.23 (1.70)	3.62 (1.98)	4.18 (1.74)	3.85 (1.76)	PFs 1, 3 > PFs 2, 4
Social capital (1–5)	4.16 (0.50)	3.80 (0.51)	4.01 (0.50)	3.90 (0.51)	PF 1 > PF 3 > PFs 2, 4
Structural resources (0–7)	2.80 (1.54)	2.47 (1.78)	2.63 (1.56)	2.33 (1.57)	PF 1 > PF 3 > PF 4
Organizational slack-space (1–5)	3.64 (1.25)	3.54 (1.25)	3.51 (1.22)	3.44 (1.20)	PF 1 > PF 3 > PF 4
Organizational slack-time (1–5)	3.65 (0.84)	3.21 (0.99)	3.39 (0.88)	3.21 (0.82)	PF 1 > PF 3 > PFs 2, 4
Organizational slack-staffing (1–5)	3.10 (1.09)	2.59 (1.16)	2.68 (1.09)	2.68 (1.09)	PF 1 > PFs 2, 3, 4
**Work-life experiences**					
Responsive behaviors from residents (0–6)	2.96 (1.71)	3.32 (1.79)	3.45 (1.63)	3.34 (1.63)	PF 1 < PFs 2, 3, 4
Rushed care (0–7)	2.38 (2.63)	3.41 (2.75)	3.46 (2.76)	3.34 (2.72)	PF 1 < PFs 2, 3, 4
Missed care (0–10)	1.18 (1.73)	1.78 (2.09)	2.03 (2.27)	1.94 (2.17)	PF 1 < PFs 2, 3, 4
Adequate job orientation (1–5)	4.27 (0.79)	4.02 (0.75)	4.11 (0.82)	4.00 (0.84)	PF 1 > PF 3 > PFs 2, 4
Job satisfaction (1–5)	4.48 (0.51)	4.00 (0.78)	4.09 (0.67)	4.02 (0.69)	PF 1 > PFs 2, 3, 4
Psychological empowerment					
Competence (1–5)	4.60 (0.45)	4.29 (0.48)	4.48 (0.46)	4.35 (0.48)	PF 1 > PF 3 > PFs 2, 4
Meaning (1–5)	4.67 (0.45)	4.29 (0.58)	4.53 (0.48)	4.38 (0.51)	PF 1 > PF 3 > PFs 2, 4
Self-determination (1–5)	4.22 (0.65)	3.76 (0.85)	4.01 (0.73)	3.80 (0.75)	PF 1 > PF 3 > PFs 2, 4
Impact (1–5)	3.86 (0.69)	3.32 (0.83)	3.76 (0.64)	3.48 (0.69)	PF 1 > PF 3 > PFs 2, 4
Work engagement					
Vigor (0–6)	5.73 (0.51)	4.49 (1.63)	5.12 (1.02)	4.78 (1.23)	PF 1 > PF 3 > PF 4 > PF 2
Dedication (0–6)	5.88 (0.31)	4.86 (1.49)	5.49 (0.77)	5.21 (0.99)	PF 1 > PF 3 > PF 4 > PF 2
Absorption (0–6)	5.92 (0.26)	5.26 (1.13)	5.74 (0.49)	5.53 (0.71)	PF 1 > PF 3 > PF 4 > PF 2
Organizational citizenship behaviors (1–5)	3.89 (0.61)	3.58 (0.70)	3.87 (0.55)	3.63 (0.59)	PFs 1, 3 > PF 4 > PF 2
**Health status**					
Physical health (0–100)	51.37 (6.82)	48.15 (7.96)	46.04 (8.48)	46.89 (8.29)	PF 1 > PFs 2, 3, 4
Mental health (0–100)	54.29 (6.78)	49.16 (10.70)	49.20 (9.40)	48.94 (9.45)	PF 1 > PFs 2, 3, 4

Notes: For each variable, the raw mean and SD are presented. Detailed descriptive statistics including skewness and kurtosis are presented in Table S3 in the Supplementary Materials. To test the difference by profile membership, we used a three-level random intercept regression analysis with the profiles as the only independent variable, controlling for clustering at facility and unit levels. Full regression results can be found in Table S4 in the Supplementary Materials. We used listwise deletion to handle missingness. Missing cases range from 0 to 11 cases for the variables examined. PF = profile. ^a^: The profile membership was the care aide’s most likely profile based on the posterior probabilities. ^b^: Bonferroni corrections used in the multiple comparisons and adjusted *p* < 0.008 were used to indicate statistical significance.

## Data Availability

The Translating Research in Elder Care (TREC) data used for this article are housed in the secure and confidential Health Research Data Repository (HRDR) in the Faculty of Nursing at the University of Alberta, in accordance with the health privacy legislation of participating TREC jurisdictions. The data were provided under specific data sharing agreements only for approved use by TREC within the HRDR. Where necessary, access to the HRDR to review the original source data may be granted to those who meet pre-specified criteria for confidential access, available at request from the TREC data unit.

## Supplementary Materials

The following supporting information can be downloaded at: https://www.mdpi.com/article/10.3390/healthcare11081145/s1, Table S1: Variables and measures; Table S2: Latent profile analysis model selection process; Table S3: Skewness and kurtosis of burnout scores, perceptions of work environment, work-life experiences, and health status. Table S4: Mean differences (regression coefficients) by profiles in perceptions of work environment, work-life experiences, and health status based on three-level random intercept linear regression.
