# Supplementary material for: The Complexity of Burnout Experiences among Care Aides: A Person-Oriented Approach to Burnout Patterns
Source: Healthcare (Basel). 2023 Apr 17;11(8):1145. doi: 10.3390/healthcare11081145 (PMC10137655; doi:10.3390/healthcare11081145)
Supplement: Supplementary file 1 [file healthcare-11-01145-s001.zip › healthcare-2205933-supplementary.pdf]

Supplementary Materials

Table S1. Variables and Measures

Variable	Description	# items	Scoring
Burnout measured with the Short Form of Maslach Burnout Inventory-General Survey [1]			
Exhaustion	Feelings of emotionally exhausted by one's job (e.g., "I feel burned out from my work")	3	Mean of the 3 items (range=0-6) measured on a 7-level scale (never-daily)
Cynicism	Feelings of cynical about and detached from work (e.g., "I have become more cynical about whether my work contributes anything")	3	Mean of the 3 items (range=0-6) measured on a 7-level scale (never-daily)
Professional efficacy	Feelings of competence and achievement at work (e.g., "I have accomplished many worthwhile things in this job")	3	Mean of the 3 items (range=0-6) measured on a 7-level scale (never-daily)
Perceptions of work environment measured with the Alberta Context Tool (ACT) [2]			
Leadership	The actions of formal leaders on the unit to influence change and excellence in practice (e.g., "the leader looks for feedback even when it is difficult to hear")	6	Mean of the 6 items (range=1-5) measured on a 5-level scale (strongly disagree-strongly agree)
Culture	The way that "we do things" on the unit; items generally reflect a supportive work culture (e.g., "I receive recognition from others about my work")	6	Mean of the 6 items (range=1-5) measured on a 5-level scale (strongly disagree-strongly agree)
Evaluation	The process of using data to assess team performances and to achieve outcomes on the unit (e.g., "I routinely receive information on my team's performance")	6	Mean of the 6 items (range=1-5) measured on a 5-level scale (strongly disagree-strongly agree)
Formal interactions	Formal exchanges that occur between care aides and other staff through scheduled activities (e.g., team meetings, change of shirt report)	4	Each item was measured on 5-level scale (never-almost always). Recode 0 = no interaction, 1 = interaction. Take count of recoded items (range=0-4)
Informal interactions	Information exchanges that occur between individuals within an organization or unit which can promote the transfer of knowledge. (e.g., hallway talk, informal bedside teaching)	9	Each item was measured on 5-level scale (never-almost always). Recode 0 = no interaction, 1 = interaction. Take count of recoded items (range=0-9)
Social capital	Active connections among people (bonding, bridging, and linking) (e.g., "people on the team share information with others on the team")	6	Mean of the 6 items (range=1-5) measured on a 5-level scale (strongly disagree-strongly agree)

Structural resources	The structural elements of an organization that facilitate the ability to assess and use knowledge (e.g., notice boards, journals in the work area, policies and procedure manuals)	7	Each item was measured on a 5-level scale (never-almost always). Recode 0 = no resource, 1 = resource. Take count of recoded items (range=0-7)
Organizational Slack- - Staffing	The cushion of actual or potential resources which allows an organization to adapt successfully to internal pressures (e.g., enough staff to deliver best possible care; adequate space to provide resident care; time to do something extra for residents)	3	Mean of the 3 items (range=1-5) measured on a 5-level scale (strongly disagree-strongly agree)
Organizational Slack - Space		2	Mean of the 2 items (range=1-5) measured on a 5-level scale (strongly disagree-strongly agree)
Organizational Slack - Time		4	Mean of the 4 items (range=1-5) measured on a 5-level scale (strongly disagree-strongly agree)
Work-life experiences			
Responsive behaviors from residents	Responsive behaviors from residents towards staff (e.g., yelling and screaming verbal threats, hurtful remarks, or behaviors)	6	Each item was measured on 0 = no and 1 = yes. Take count of recoded items (range=0-6)
Rushed care	Self-reported care tasks that were rushed during respondent's most recent shift (e.g., taking residents for a walk, talking with residents, mouth care)	7	Each item was measured on 0 = no and 1 = yes. Take count of recoded items (range=0-7)
Missed care	Self-reported care tasks that were left undone during the respondent's most recent shift (e.g., taking residents for a walk, talking with residents, mouth care)	10	Each item was measured on 0 = no and 1 = yes. Take count of recoded items (range=0-10)
Adequate job orientation	Getting sufficient orientation when starting a new job	1	5-level scale (1=strongly disagree-5=strongly agree)
Job Satisfaction	A global measure of job satisfaction (e.g., "all in all, I am satisfied with my job") adapted from the Michigan Organizational Assessment Questionnaire Job Satisfaction Subscale (MOAQ-JSS-3) [3].	3	Mean of the 3 items (range=1-5) measured on a 5-level scale (strongly disagree-strongly agree)
Phycological Empowerment (PE) measured with an adapted version of Spreitzer's Psychological Empowerment Measure [4]			
PE Competence	A belief in one's capability to perform work activities with skill (e.g., "I have all the skills needed to do my job well")	3	Mean of the 3 items (range=1-5) measured on a 5-level scale (strongly disagree-strongly agree)

PE Meaning	A fit between the needs of one's work role and one's beliefs, values, and behaviors (e.g., "The work I do is meaningful to me")	3	Mean of the 3 items (range=1-5) measured on a 5-level scale (strongly disagree-strongly agree)
PE Self-determination	A sense of autonomy or choice over the initiation and continuation of work behavior and processes (e.g., "I have the flexibility to decide how to do my job")	3	Mean of the 3 items (range=1-5) measured on a 5-level scale (strongly disagree-strongly agree)
PE Impact	The degree to which one can influence strategic, administrative, or operating outcomes at work (e.g., "my work makes a big difference on my unit")	3	Mean of the 3 items (range=1-5) measured on a 5-level scale (strongly disagree-strongly agree)
Work Engagement measured with an adapted version of the Utrecht Work Engagement Scale-9 items (UWES) [5]			
UWES Vigor	High levels of energy and resilience, the willingness to invest effort in one's job (e.g., "at my job, I feel strong and vigorous")	3	Mean of the 3 items (range=0-6) measured on a 7-level scale (never-daily)
UWES Dedication	A strong involvement in one's work, accompanied by feelings of enthusiasm and significance (e.g., "I am excited about doing my job")	3	Mean of the 3 items (range=0-6) measured on a 7-level scale (never-daily)
UWES Absorption	A pleasant state of total immersion in one's work (e.g., "when I am working, all of my attention is on my work")	3	Mean of the 3 items (range=0-6) measured on a 7-level scale (never-daily)
Change-oriented organizational citizenship behaviors [6]	An individual's efforts to identify and implement changes on work methods, policies, and procedures to improve the situation and performance (e.g., "I often suggest ways to improve rules or policies")	4	Mean of the 4 items (range=1-5) measured on a 5-level scale (strongly disagree-strongly agree)
Health status measured with the Short Form 8 Health Survey (SF-8) [7]			
SF-8 Physical health	Respondent perception of physical health in last 4 weeks	8	Scoring of items on 5 or 6-level scales and computed using the weightings provided by the scale developers (range=0-100)
SF-8 Mental health	Respondent perception of mental health in last 4 weeks		

Table S2. Latent Profile Analysis Model Selection Process

Decision-making in selecting the best-fit model based on predefined model selection criteria:<br>Criteria used in the model selection process include Akaike information criterion (AIC), Bayesian Information Criterion (BIC), sample-size adjusted BIC, Lo, Mendell, and Rubin (LMR) test, and entropy (measuring classification uncertainty). We also considered conceptual meaning, interpretability of profiles, model parsimony in the model selection process.<br><br>The information criteria, including AIC, BIC, and sample-size adjusted BIC, indicated that models with a higher number of profiles had a better fit of the data. However, the improvement in model fit shrank after the 6-profile model with a small decrease in the information criteria from the 6-profile model to the 7-profile model. All models, except for the 3-profile model, had an entropy value of .80 or greater, a threshold value representing minimal classification uncertainty. Although the information criteria and entropy support the 6-profile model, this model had a profile that contained 1% of the sample, a spurious profile with little theoretical and practical meaning. The 6-profile model was therefore rejected. The LRM test that compared the 5-profile model to the 4-profile model suggested that an addition of one more profile did not significantly improve the model fit and therefore supported the 4-profile model. The 4-profile model contains a profile that accounts for approximately 3% of the sample. Since this small profile also existed in both the 5-profile and 6-profile models, we considered it represented a consistent group of care aides that deserved further investigation. As a result, the 4-profile model was retained as the final model for the post hoc analyses.
Sensitivity analysis:<br>We reran latent profile analysis using updated composite scores of Cynicism and Professional Efficacy where we excluded the following items that had standardized factors loadings in a confirmatory factor analysis lower than 0.5:<br>• Cynicism item "I just want to do my job and not be bothered" with factor loading=0.38<br>• Professional Efficacy item "In my opinion, I am good at my job" with factor loading=0.36<br>The results based on the updated scores were consistent with the original results. Nevertheless, we acknowledged the weaknesses of using MBI composite scores, such as the risk of potentially mixing/combining causally separate entities which can result in unclear patterns and weak/small differences between patterns. In this regard, we encourage future studies to attempt a variety of person-centered analysis methods such as mixture factor analysis that uses validated individual items instead of composite scores of scales as indicators.<br><br>We also replicated the latent profile analysis with an earlier wave of the TREC care aide survey (collected between September 2014 and May 2015) and identified similar patterns.

Table S3 Skewness and Kurtosis of Burnout Scores, Perceptions of Work Environment, Work-Life Experiences, and Health Status

	Skewness	Kurtosis
Burnout		
Exhaustion	0.01	1.96
Cynicism	0.09	2.15
Professional efficacy	-1.74	6 45
Perceptions of work environment		
Leadership	-0.54	4.19
Culture	-0.43	3.62
Evaluation	-0.72	4.46
Formal interactions		
Informal interactions	0.18	2.79
Social capital	-0.31	3.88
Structural resources	0.22	2.24
OS-Space	-0.73	2.18
OS-Time	-0.10	2.39
OS-Staffing	-0.01	2.04
Work-life experiences		
Responsive behaviors from residents	-0.34	2.33
Rushed care	0.25	1.45
Missed care	1.76	6.49
Adequate job orientation		
Job Satisfaction	-0.84	4.40
Empowerment		
Competence	-0.28	1.75
Meaning	-0.52	2.27
Determination	-0.80	4.07
Impact	-0.21	3.00
Work Engagement		
Vigor	-2.09	8.11
Dedication	-2.78	12.56
Absorption	-3.79	19.19
Organizational citizenship behaviors	-0.41	3.84
Health status		
Physical health	-0.85	3.44
Mental health	-1.29	4.62

Table S4. Mean Differences (Regression coefficients) by Profiles in Perceptions of Work Environment, Work-Life Experiences, and Health Status Based on Three-Level Random Intercept Linear Regression[a]

	Mean difference compared to Profile 1 Engaged			Mean difference compared to Profile 3 Overwhelmed/accomplished		Mean difference compared to Profile 4 Tired but effective
	Profile 2 Tired and ineffective	Profile 3 Overwhelmed but accomplished	Profile 4 Tired but effective	Profile 2 Tired and ineffective	Profile 4 Tired but effective	Profile 2 Tired and ineffective
Perceptions of work environment						
Leadership	-0.38 ǂ	-0.16 ǂ	-0.23 ǂ	-0.22 ǂ	-0.07 *	-0.15 *
Culture	-0.36 ǂ	-0.19 ǂ	-0.27 ǂ	-0.17 ǂ	-0.08 ǂ	-0.09
Evaluation	-0.28 ǂ	-0.11 ǂ	-0.24 ǂ	-0.17 ǂ	-0.13 ǂ	-0.04
Formal interactions	-0.04	0.01	-0.10 ǂ	-0.06	-0.12 ǂ	0.06
Informal interactions	-0.61 ǂ	-0.03	-0.36 ǂ	-0.58 ǂ	-0.32 ǂ	-0.26
Social capital	-0.36 ǂ	-0.15 ǂ	-0.27 ǂ	-0.22 ǂ	-0.12 ǂ	-0.09
Structural resources	-0.34 *	-0.20 ǂ	-0.49 ǂ	-0.13	-0.28 ǂ	0.15
OS-Space	-0.12	-0.12 ǂ	-0.26 ǂ	0.00	-0.14 ǂ	0.14
OS-Time	-0.43 ǂ	-0.27 ǂ	-0.44 ǂ	-0.16	-0.16 ǂ	0.00
OS-Staffing	-0.47 ǂ	-0.42 ǂ	-0.38 ǂ	-0.05	0.04	-0.09
Work-life experiences of						
Responsive behaviors from residents	0.39 *	0.49 ǂ	0.35 ǂ	-0.10	-0.14	0.04
Rushed care	0.96 ǂ	1.14 ǂ	0.90 ǂ	-0.18	-0.24	0.06
Missed care	0.52 *	0.85 ǂ	0.70 ǂ	-0.34	-0.15	-0.18
Adequate job orientation	-0.26 ǂ	-0.14 ǂ	-0.28 ǂ	-0.11	-0.13 ǂ	0.02
Job Satisfaction	-0.48 ǂ	-0.38 ǂ	-0.46 ǂ	-0.10	-0.07 *	-0.02
Empowerment						
Competence	-0.31 ǂ	-0.12 ǂ	-0.25 ǂ	-0.19 ǂ	-0.13 ǂ	-0.07
Meaning	-0.38 ǂ	-0.14 ǂ	-0.30 ǂ	-0.24 ǂ	-0.15 ǂ	-0.08
Determination	-0.46 ǂ	-0.21 ǂ	-0.41 ǂ	-0.25 ǂ	-0.20 ǂ	-0.05
Impact	-0.55 ǂ	-0.10 ǂ	-0.37 ǂ	-0.44 ǂ	-0.27 ǂ	-0.17 *
Work Engagement						
Vigor	-1.22 ǂ	-0.61 ǂ	-0.93 ǂ	-0.61 ǂ	-0.32 ǂ	-0.29 ǂ
Dedication	-1.01 ǂ	-0.39 ǂ	-0.67 ǂ	-0.62 ǂ	-0.27 ǂ	-0.35 ǂ
Absorption	-0.66 ǂ	-0.19 ǂ	-0.39 ǂ	-0.48 ǂ	-0.20 ǂ	-0.27 ǂ
Organizational citizenship behaviors	-0.31 ǂ	-0.02	-0.26 ǂ	-0.29 ǂ	-0.24 ǂ	-0.06
Health status						
Physical health	-3.21 ǂ	-5.36 ǂ	-4.45 ǂ	2.15 *	0.91 *	1.24
Mental health	-5.07 ǂ	-5.21 ǂ	-5.29 ǂ	0.14	-0.09	0.22

Notes. OS=organizational slack.

a: A three-level random intercept regression analysis is estimated for each variable with the profiles as the only independent variable controlling for clustering at facility and unit levels. For each variable three sets of models where the reference group was profile 1, 3, 4, respectively.
ǂ $p<0.008$ (Bonferroni-corrected p-value given 6 comparisons are involved)
* $p<0.05$

References

1. Maslach, C.; Jackson, S.E.; Leiter, M.P.; Schaufeli, W.B.; Schwab, R.L. *Maslach burnout inventory*; Consulting psychologists press Palo Alto, CA: 1986; Volume 21.
2. Estabrooks, C.A.; Squires, J.E.; Hayduk, L.A.; Cummings, G.G.; Norton, P.G. Advancing the argument for validity of the Alberta Context Tool with healthcare aides in residential long-term care. *BMC Med Res Methodol* **2011**, *11*, 107, doi:10.1186/1471-2288-11-107.
3. Bowling, N.A.; Hammond, G.D. A meta-analytic examination of the construct validity of the Michigan Organizational Assessment Questionnaire Job Satisfaction Subscale. *J Vocat Behav* **2008**, *73*, 63-77, doi:DOI 10.1016/j.jvb.2008.01.004.
4. Spreitzer, G.M. Psychological empowerment in the workplace: Dimensions, measurement, and validation. *Academy of management Journal* **1995**, *38*, 1442-1465.
5. Schaufeli, W.B.; Bakker, A.B.; Salanova, M. The measurement of work engagement with a short questionnaire: A cross-national study. *Educational and psychological measurement* **2006**, *66*, 701-716.
6. Choi, J.N. Change - oriented organizational citizenship behavior: effects of work environment characteristics and intervening psychological processes. *Journal of Organizational Behavior: The International Journal of Industrial, Occupational and Organizational Psychology and Behavior* **2007**, *28*, 467-484.
7. Ware, J.E.; Kosinski, M.; Dewey, J.E.; Gandek, B. How to score and interpret single-item health status measures: a manual for users of the SF-8 health survey. *Lincoln, RI: QualityMetric Incorporated* **2001**, *15*, 5.
